# Mu rhythm desynchronization reveals motoric influences of hand action on object recognition

**DOI:** 10.3389/fnhum.2013.00066

**Published:** 2013-03-07

**Authors:** Sanjay Kumar, M. J. Riddoch, Glyn Humphreys

**Affiliations:** Department of Experimental Psychology, Oxford UniversityOxford, UK

**Keywords:** affordance perception, object recognition, mu rhythm suppression, EEG, cognition

## Abstract

We examined the effect of hand grip on object recognition by studying the modulation of the mu rhythm when participants made object decisions to objects and non-objects shown with congruent or incongruent hand-grip actions. Despite the grip responses being irrelevant to the task, mu rhythm activity on the scalp over motor and pre-motor cortex was sensitive to the congruency of the hand grip—in particular the event-related desynchronization of the mu rhythm was more pronounced for familiar objects grasped with an appropriate grip than for objects given an inappropriate grasp. Also the power of mu activity correlated with RTs to congruently gripped objects. The results suggest that familiar motor responses evoked by the appropriateness of a hand grip facilitate recognition responses to objects.

## Introduction

There is a growing body of evidence indicating that the visual system responds to action possibilities in an image (to “visual affordance”; see Gibson, 1979). For example, Tucker and Ellis (1998) showed that the time to make upright or inverted decision to objects using the left or right hand is affected by the orientation of the handle depicted in the image. Responses are faster when the orientation of the handle is congruent with the hand used for the response. Such congruency effects are suggestive that motor responses are automatically activated by objects, and this influences the speed of responding (which is faster when the activated motor response matches the response for the task). Other research indicates that it is not only the properties of objects, but also the way they interact with body parts that “affords” action. Yoon and Humphreys (2005) had participants verify a name to an object that was depicted with a hand offering a grip that was either congruent or incongruent with the action applied to use the object. Although the grip was irrelevant to the verification task they found that responses were affected by the congruency of the hand grip. Placing objects in relation to the hands also influences object classification. Yoon et al. (2010) had participants classify pairs of objects on the basis of whether they would normally be used together. The objects were presented either alone or alongside a stooge whose hands reached to each object. Classification responses were faster when the objects were presented in their normal co-locations for action (e.g., fork on the left, knife on the right), and this effect of object positioned was particularly strong when the stimuli were aligned with the arms of the stooge. Yoon et al. propose that the possibility of action, evoked by placing objects in correct positions in relation to the body, enhances object classification. There are also effects apparently evoked directly by seeing the hand adopt a particular grip. Borghi et al. (2005) showed that categorical decisions to manipulable artifacts vs. natural objects were affected when photographs of hand postures with a power or precision grip were used as primes. For example, participants were faster to respond to natural objects which could be grasped by a precision grip when the prime was a precision grip hand posture. The results are consistent with responses to objects being primed by the pre-activation of a motor response, triggered by the hand grasp.

The factors critical for these effects of body stimuli on responses to objects, however, have yet to be fully specified. In an fMRI study with objects positioned for action similar to those of Yoon et al. (2010), Roberts and Humphreys (2010) found increased brain activity in visual brain regions (the lateral occipital complex and anterior fusiform gyrus) for objects shown in action-related vs. unrelated positions. These data suggest that part of the action-based effects may reflect enhanced visual processing, perhaps because interacting objects are visually familiar. One possibility, then, is that the sight of body parts interacting with objects leads to a similar “direct” enhancement of visual processing. A further possibility, though, is that the body parts evoke a motor response that is modulated by whether the objects are gripped appropriately or inappropriately for action. An enhanced motor response to a congruently gripped object may lead to faster classification times. EEG data are consistent with this. Kumar et al. (2012) presented images of manipulable objects with congruent and incongruent grips while recording EEG responses. Congruently gripped objects generated an early enhanced response over motor cortex in the P1 time window (90–120 ms) and over posterior brain areas in the later N1 time window (130–150 ms). The data suggest that congruently gripped objects evoke a rapid motor response, which may feedback to enhance object processing. There was also evidence for facilitated motor planning of the response to congruently gripped objects, reflected in the lateralized readiness potential. At a later time period (after 180 ms) Petit et al. (2006) have reported increased neuronal responses over motor cortex for objects depicted with an awkward grasp, perhaps then reflecting the difficulty of using the object. These rapid motor responses to objects may stem from so-called canonical neurons (neurons associated with visuo-motor transformations of objects) which are activated when a hand shapes to grasp an object (Fogassi et al., 2001). On the other hand, the “classic” mirror neuron system appears not to be sensitive to how objects are grasped (Johnson-Frey et al., 2003).

In the present study we present converging evidence for the involvement of rapidly-evoked motor responses to correctly gripped objects using EEG-based oscillatory activity. We analysed event-related desynchronization/synchronization (ERD/ERS) of the EEG response to objects shown with congruent or incongruent grips. ERD can be used as an index of neural excitation (Goldman et al., 2002) whereas ERS reflects an inactive network state (Pfurtscheller et al., 1996). Most notably, ERD observed in the mu frequency band (8–12 Hz) is typically taken as evidence of motor preparation (Pfurtscheller et al., 1997, 2000; Derambure et al., 1999; Pineda, 2005) and has been observed in relation to both object-directed grasp responses (Pfurtscheller et al., 1996; Muthukumaraswamy and Johnson, 2004) and precision grips (Muthukumaraswamy et al., 2004). Here we assessed evidence for increased ERD in the mu frequency band over scalp motor regions when participants made object decisions to congruently and incongruently gripped objects, and whether this related to behavioral performance. Evidence for changes in mu activity would fit with there being early-evoked motor responses to objects that are mediated by grip congruency.

## Methods

### Participants

Seventeen (3 male) undergraduate students of the School of Psychology, University of Birmingham, participated for cash or course credit. All the subjects were right handed (self report) and had normal or corrected-to-normal vision. Participants provided written consent prior to participation. The study was approved by the Local Ethics Committee of the University of Birmingham and conformed to the Declaration of Helsinki.

#### Materials

**Figure 1:**
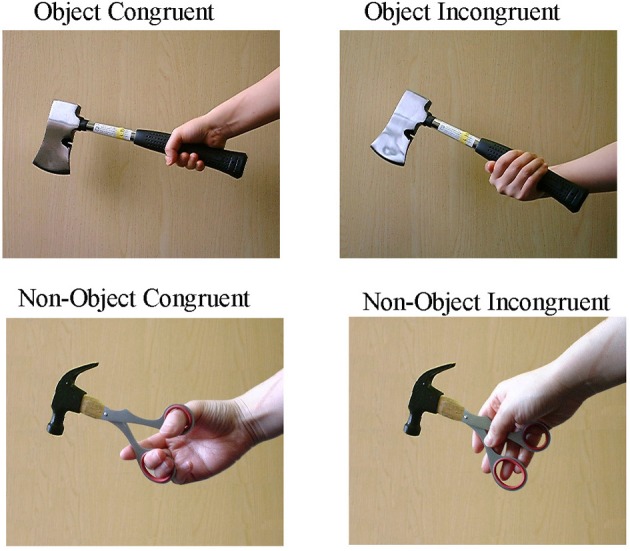
**Examples of the stimuli used in the experiment.** Objects and non-objects were gripped congruently or incongruently.

The stimuli were 2D pictures based on 30 graspable real objects. Based on these real objects, 30 graspable non-objects were produced in Adobe Photoshop CS by combining the parts of two different objects. The images of real objects were paired pseudo-randomly and from each pair a handle of one object and the main “body part” of another object were extracted and merged together to generate a non-object (see Figure 1 for example stimuli). The non-objects were all visually inspected and judged to be “usable.” Every object was photographed with a congruent grip and an incongruent grip, and every non-object was edited to include either a congruent or an incongruent grip depending on the relations between the hand and the handle. In the incongruent grip condition, a grip was chosen that was appropriate for another real object, so that congruent and incongruent grips did not differ in their visual properties across the complete set of stimuli. The frame size of the stimuli was 450 pixels wide and 370 pixels high (degree of visual angle: 10°), and this window was placed at the center of the computer screen throughout the experiment.

#### Design and procedure

Participants were required to ignore the depicted hand-grips and to focus on the objects and non-objects. The task was to decide as quickly as possible whether the target was a real object or a non-object. Participants responded by pressing the keys on the keyboard with either their right or left hand index fingers (nine participants used their right had to respond “yes,” the other eight used their left hand). The order of the tasks and the assignment of the left and right keys to the “yes” and “no” responses were counterbalanced across participants. Participants received 120 stimuli; 30 objects and the same number of non-objects and each was depicted with a congruent hand grip or an incongruent handgrip.

**Figure 2:**
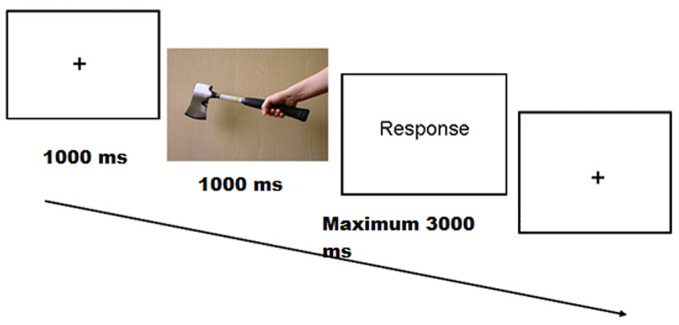
**Trial structure used in the experiment.** In this example the stimulus is in the congruent grip condition.

The participants received 12 practice trials before each task. Each trial began with the presentation of a fixation point for 1000 ms in the middle of the screen, which was followed by a target stimulus for 1000 ms. Participants had to make a response as quickly and accurately as possible and within a deadline of 4000 ms after stimulus onset (Figure 2 shows a typical trial presentation). Online electroencephalograms (EEGs) were measured while participants performed the task.

### EEG recording and data processing

EEG was recorded continuously with Ag/AgCl electrodes from 128 scalp electrode locations. The electrodes were placed according to the 10-5 electrode system (Oostenveld and Praamstra, 2001) using a nylon electrode cap. Vertical eye movements were monitored through an electrode placed on the left eye infra-orbital region and horizontal by bipolar electrodes placed on the outer canthi of each eye. Common Mode Sense (CMS) and Driven Right Leg (DRL) electrodes were used as references and ground. EEG and electro-occulogram (EOG) signals were amplified with BioSemi Active-Two amplifiers and sampled at 1024 Hz. The continuous EEG recordings were off-line referenced to average of left and right mastoids. Eye movement correction was done using a regression based method implemented in Brain Vision Analyser (Gratton et al., 1983). Continuous EEG was segmented in epochs from 1000 ms before target-onset to 1000 ms after target-onset. Activity for 1000 ms pre-stimulus was taken as the reference interval and reflected activities associated with fixation cross processing. Epochs were discarded if the voltage exceeded ±100 μ volt. The remaining epochs were band pass filtered in narrow frequency band of 8–10 Hz and 10–12 Hz (24 db/oct) for further analysis. We chose two frequency bands of 8–10 and 10–12 which may reflect (i) widespread non-specific movement and (ii) focused specific movement activities, respectively (Pfurtscheller et al., 2000). ERD/ERS were computed according to the commonly used approach (Pfurtscheller and Aranibar, 1977, 1979; Pfurtscheller and Neuper, 1994; Pineda, 2005). Bandpass filtered epoch's amplitude were squared and averaged across all trials for each conditions separately. For each data point, ERD/ERS were calculated in accordance with the standard formula: [(band-power-active-interval − band-power-reference-interval)/band-power-reference-interval] × 100. Smoothing of ERD/ERS traces was performed by using a moving averaging window of 100 ms. ERD/ERS was calculated on pooled 8 electrodes from each hemisphere representing scalp activity over primary sensory motor (PSM) region and for supplementary motor area (SMA) by pooling 4 central electrodes as reported in an earlier high density EEG study of alpha ERD (Babiloni et al., 1999). ERD/ERS was calculated for mean activity in every 100 ms time window after stimulus onset on the smoothed ERD/ERS traces.

## Results

### Behavioral results

**Figure 3:**
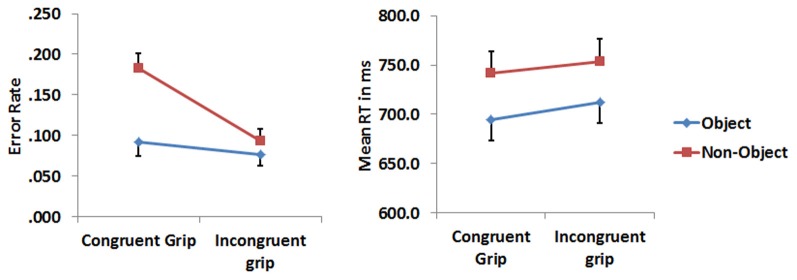
**The error rate and RTs related to congruently and incongruently gripped objects and non-objects.** The error bars represent 1 standard error.

Error rates and median reaction times (RTs) for correct response trials were analysed with a 2 (object type) × 2 (grip) repeated measure analysis of variance (RMANOVA). Paired *t*-tests were used to decompose the interactions. RTs to objects were significantly faster compared to those to non-objects [*F*_(1, 16)_ = 11.955, *p* = 0.003]. Neither the main effect of grip nor the grip × object type interactions were significant (all *F*_s_ < 1). For errors there were main effects reflected reduced error rates for objects compared to non-objects [*F*_(1, 16)_ = 7.001, *p* = 0.017] and to stimuli with incongruent relative to congruent grips [*F*_(1, 16)_ = 22.162, *p* = 0.001]. There was also a significant interaction between object type and grip [*F*_(1, 16)_ = 12.161, *p* = 0.003]. Participants made more errors when classifying non-objects with a congruent grip compared with non-objects with an incongruent grip (*t* = 5.190, *p* = 0.001). Their accuracy was also worse for non-objects gripped congruently than objects gripped congruently (*t* = 3.756, *p* = 0.002). Figure 3 depicts median RTs and the error rate.

### Topographic analysis

**Figure 4:**
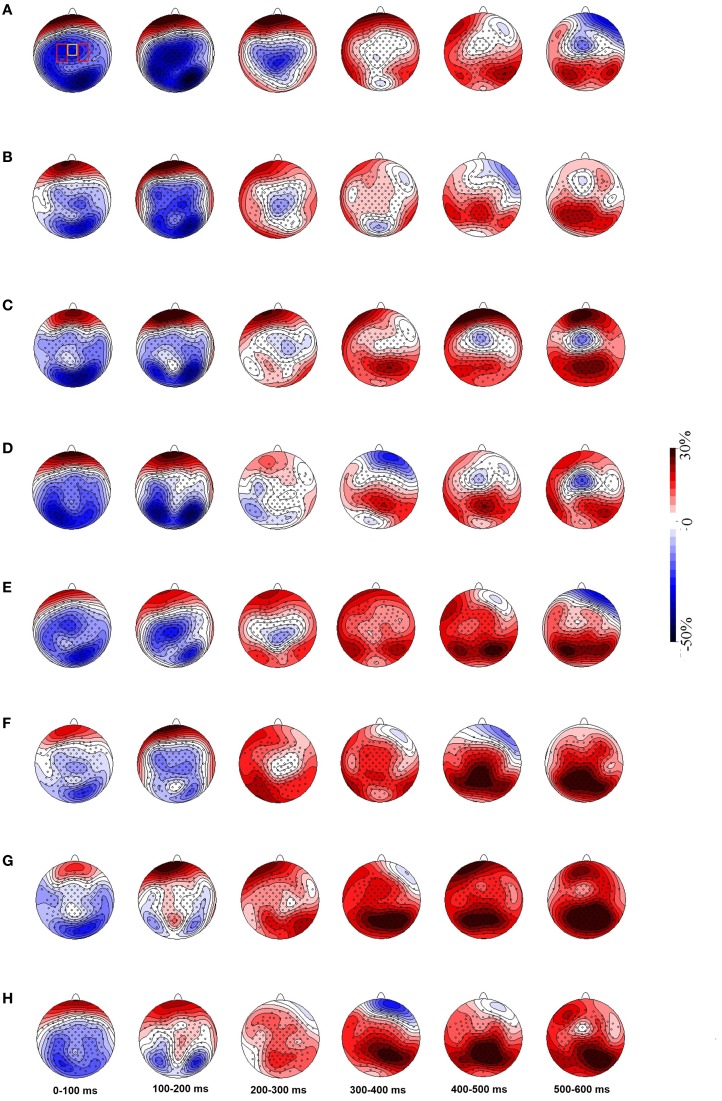
**Grand averaged topographic maps show ERD/ERS in 8–10 Hz (A–D) and 10–12 Hz (E–H) mu frequency bands.** Panels **(A)** and **(E)** represent the object congruent grip condition, **(B)** and **(F)** the object incongruent grip condition, **(C)** and **(G)** the non-object congruent grip condition and **(D)** and **(H)** the non-object incongruent grip condition. Electrodes pooled over the PSM areas are shown in red rectangles and yellow rectangles show electrodes pooled over the SMA. Panel **(A)** reflects the condition with congruent grips to objects and shows an extended period of ERD activation. ERDs are followed by ERS in later time windows. ERD, event-related desynchronization; ERS, event-related synchronization; PSM, primary sensory motor area, SMA, supplementary motor area.

Topographic maps of ERD/ERS activity, created by spherical spline interpolation, showed mu rhythm ERD across electrodes over motor cortex lasting around 300 ms after stimulus onset. We also observed what was likely alpha ERD in the same time window across the posterior brain areas which may reflects sensory processing of the stimuli (Figure 4). Alpha and mu have overlapping frequency distributions but are functionally different. Figure 4 also shows that ERD in the non-object conditions over motor cortex was shorter and weaker than that found in the object conditions.

### ERD/ERS analysis

Statistical analysis of activity in electrodes over PSM scalp regions was carried out using a RMANOVA with 2 (hemispheres) × 2 (object-non-object) × 2 (grip) factors. A similar analysis was conducted on activity over the SMA scalp region with 2 (objects) × 2 (grip) factors, with SMA activity computed from one pooled area over the central brain region.

**Figure 5:**
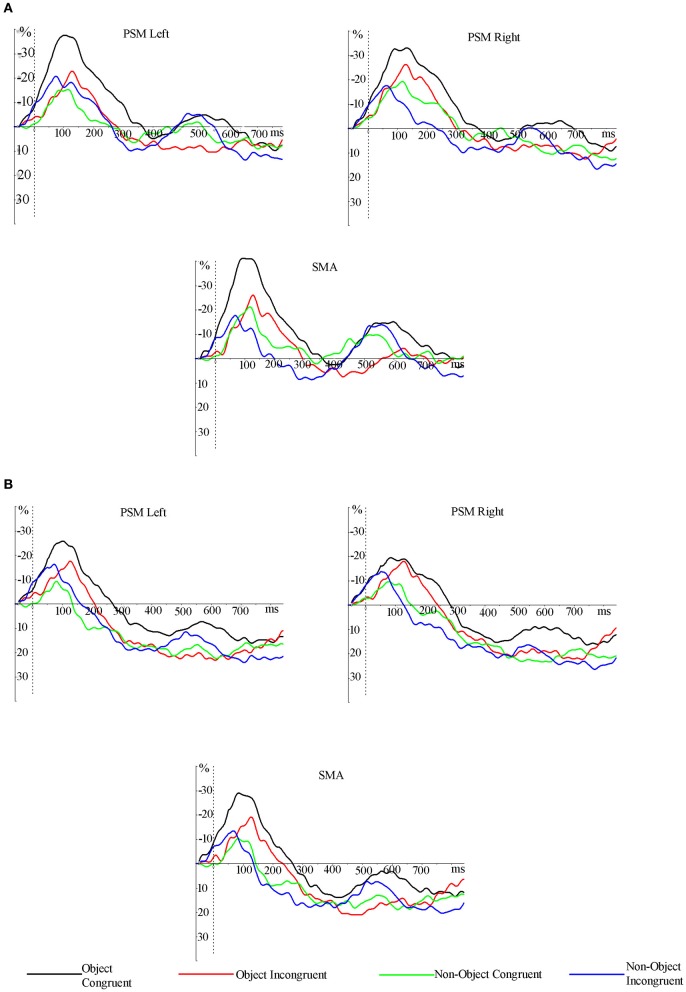
**Grand averaged ERD/ERS traces (smoothed across a 100 ms moving window) from electrodes pooled across the scalp regions of primary sensory motor area and supplementary motor area (PSM and SMA).** ERS for non-objects started earlier than for objects and ERD related to congruently gripped objects lasted longer and had a greater amplitude in the lower **(A)** and the upper **(B)** mu frequency bands. ERD, event-related desynchronization; ERS, event-related synchronization; PSM, primary sensory motor area; SMA, supplementary motor area.

A significant 3-way interaction [hemisphere × object type × grip; *F*_(1, 16)_ = 8.125, *p* = 0.012] and main effect of object type [object > non-object; *F*_(1, 16)_ = 4.713, *p* = 0.045] was observed in the 100–200 ms time window in the 8–10 Hz mu band. The data presented in Figures 4 and 5 indicate that ERD was higher in the left hemisphere for congruently gripped objects, compared with the other conditions. Breakdown of the interaction effect was carried out by analysing activity in the left and right hemispheres separately. Taking activity in the right hemisphere only, the ERD for objects was reliably higher than for non-objects [*F*_(1, 16)_ = 6.370, *p* = 0.023]. However, there were no reliable effects of grip [*F*_(1, 16)_ = 1.088, *p* = 0.312] and no interactions [*F*_(1, 16)_ = 0.628, *p* = 0.440]. For the left hemisphere there was an interaction of object type and grip [*F*_(1, 16)_ = 7.536, *p* = 0.014]. There was greater power in the mu band for congruently gripped objects relative to congruently gripped non-objects (*t* = 2.486, *p* = 0.024) and (marginally) relative to incongruently gripped objects (*t* = 2.061, *p* = 0.056).

In the same mu band and time period significantly higher ERD was also observed for objects compared to non-objects over the SMA [*F*_(1, 16)_ = 5.207, *p* = 0.037].

Across the same time window (100–200 ms) ERD/ERS activity across the PSM in the upper mu band also showed a significant three-way interaction [*F*_(1, 16)_ = 9.807, *p* = 0.006] along with significantly higher ERD for objects than non-objects (main effect: *F*_(1, 16)_ = 6.088, *p* = 0.025). In the right hemisphere there was a reliable effect of object type (object > non-object; *F*_(1, 16)_ = 6.717, *p* = 0.020) but no effect of grip and no interaction (all *F*_s_ < 1). In the left hemisphere there was a marginal object type × grip interaction [*F*_(1, 16)_ = 3.242, *p* = 0.091], with congruently gripped objects having more ERD power than congruently gripped non-objects condition (*t* = 2.489, *p* = 0.024). There was a significant main effect of object type, with higher ERD power for objects than non-objects [*F*_(1, 16)_ = 4.594, *p* = 0.048]. Over the SMA the upper mu rhythm ERD was significantly higher for objects than non-objects across the same time period [*F*_(1, 16)_ = 6.310, *p* = 0.025].

After 200 ms, the ERS started to emerge mainly for non-objects and incongruently gripped objects in the mu frequency bands, and this continued until at least 300 ms post-stimulus onset (Figures 4 and 5A,B). These data were not analysed further as they were not the focus of the present paper.

We also tested for effects of the conditions in the alpha frequency band over occipital areas. For this analysis we pooled activity from four electrodes over occipital scalp regions (O1, POO9h, OI1h, PO3: O2, POO10h, OI2h, PO4h) from the left and right hemispheres. None of the effects were reliable.

### Relations to behavior

**Figure 6:**
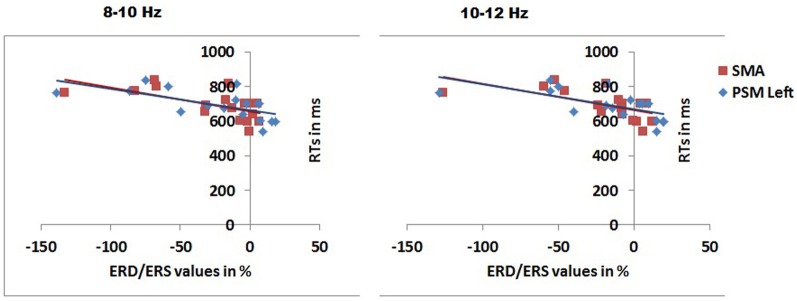
**Scatter plots with the best fitting linear lines showing significant correlations between RTs for congruently gripped objects and mu rhythm ERD/ERS over different scalp regions in 8–10 and 10–12 Hz frequency band in the 0–100 ms time window after stimulus presentation.** PSM, primary sensory motor area; SMA, supplementary motor area.

We also examined correlations between the EEG data and behavior, using Pearson product moment correlations and Bonferroni correction for multiple comparisons. In the 0–100 ms time window and 8–10 Hz range there were reliable negative correlations between RTs to congruent objects and the mu ERD in the same condition, for the left PSM (*r* = −0.634, *p* = 0.006) and the SMA (*r* = −0.628, *p* = 0.007). The same correlations were also reliable in the 10–12 Hz range (left PSM, *r* = −0.645, *p* = 0.005; SMA *r* = −0.626, *p* = 0.007). Figure 6 shows the correlations.

## Discussion

We examined the effects of depicting objects with a congruent or incongruent hand grip on brain activity in the mu rhythm over brain regions involved in motor programming and enactment. Even though the hand grip was irrelevant to the object decision task, it significantly affected performance. Participants responded faster to objects than to non-objects and there were reliable effects on errors too- non-objects depicted with a congruent hand grip were difficult to reject as non-objects. This latter result likely reflects a mismatch between the grip (congruent with a potential action) and the stimulus (a non-object), with participants making errors due to classifying the stimulus on the basis of the action depicted rather than the form. Given that grip was irrelevant to the task, and that grip congruence disrupted performance to non-objects, then the data indicate that effects of object grasp are difficult to ignore and can automatically affect object discrimination. The behavioral results observed in our study supports previous findings such as those of Borghi et al. (2005, 2007) who showed that the presence of a congruent grasp prior to the an object affected the time to decide whether the object was an artifact or natural stimulus (see also Helbig et al., 2006, 2010; Vainio et al., 2008). In addition, there were significant effects of object grip on electrophysiological activity, with early modulation of ERD in the mu band. Most notably, objects assigned a correct grip showed enhanced and prolonged ERD over primary motor cortex scalp region and SMA scalp regions, when compared to the other conditions (objects assigned an incongruent grip or non-objects). These effects emerged within a time window between 100 and 200 ms after stimulus onset. Furthermore, the ERD was higher across the PSM region for congruently gripped objects in both the lower and upper mu bands between 100 and 200 ms. Correlations between ERD activity for congruent hand grips to objects and RTs in that condition emerged over the left hemisphere sites across an even earlier time window, linking the ERD effects to behavior.

The enhanced mu rhythm we found in the left hemisphere when objects were gripped congruently may indicate the early activation of a motor response to these stimuli. Previous research has shown that the left hemisphere is dominant for the representation and planning of motor action (Haaland and Harrington, 1996; Rushworth et al., 2001). In a recent EEG study, Proverbio et al. (2011) also showed that brain responses related to tools were stronger in the left hemisphere and there is considerable evidence for left lateralization of deficits in tool use in apraxia (Kalenine et al., 2010) and in fMRI in normal participants (Króliczak and Frey, 2009). In the current data the effects of congruent grip modulated mu rhythm in both upper and lower frequency bands. Previous work indicates a functional distinction between lower (8–10 Hz) and upper (10–12 Hz) mu rhythm activity, associated respectively, with widespread non-specific movement and focused specific movement activities (Pfurtscheller et al., 2000). The advantage for congruent grips that we report was present across both frequency bands, consistent with both non-specific and specific movements being activated.

In general our results are compatible for a broad set of other data. In a recent EEG study examining power changes in mu rhythm, Proverbio (2012) found decreased power for manipulable objects compared to non-manipulable objects in 10–12 Hz frequency band over centro-parietal scalp regions. Perry and Bentin (2009) have also shown that mu rhythm desynchronization is larger when a hand grasps an object compared to when repetitive hand movements are made. Likewise Muthukumaraswamy et al. (2004) found that mu rhythm was more suppressed when participants grasped an object compared to when a grasp was not object-directed. Goal-directed activities have also been shown to modulate mu rhythms more than non-goal directed activity (Babiloni et al., 1999). Here we propose that early mu rhythm de-synchronization to congruently gripped objects reflects activation of a goal-based action to grasp the depicted object.

The current results support our prior findings which demonstrated an effect of hand grip on object decisions in early ERP components over motor cortex (P1), followed by later effects over more posterior brain regions (N1). In addition, we earlier reported effects on motor preparation (modulation of lateralized readiness potentials). The results are consistent with congruent hand grip generating a rapid and relatively automatic motor response to objects, especially when a familiar object is presented. This enhanced motor response may both feedback to modulate visual processing (Kumar et al., 2012) and prepare a more rapid response to congruently gripped objects.

Our finding that mu rhythm de-synchronization correlated with object decision responses also matches previous findings such as those of Borghi et al. (2005, 2007) who found that the presence of a congruent grasp presented prior to an object affected semantic decisions as to whether an object was an artifact or a natural stimulus (see also Helbig et al., 2006, 2010; Vainio et al., 2008). Our ERD data suggest that objects assigned a congruent grip evoke an enhanced motor response independent of lower-level sensory changes associated with applying a congruent grip to the objects (note that there was no effect on occipital alpha activity). The early ERD effect to congruently gripped objects indicates in turn that the motor system is tuned to familiar body responses to objects, enabling motor preparation to be rapidly triggered in relation to the appropriate visual cue. The data fit with “dual-route” accounts of visually-evoked action, which assume that visual cues can provide an associative trigger to the motor system independently of access to semantic knowledge (see Riddoch et al., 1989; Yoon et al., 2002). Such triggers are provided by familiar objects more than non-objects. Previous work indicates that mu rhythms are affected more by goal directed activities than non-goal directed action (Babiloni et al., 1999). Here we suggest that sight of the congruently gripped object primed participants to respond with a goal directed action to familiar objects. This triggered action also linked to the speed of the behavioral response to congruently gripped stimuli, perhaps because the behavioral response was associated with responding to a familiar object. In contrast, any motor action triggered by a congruently gripped non-object may disrupt responding to the stimulus as a non-object, and indeed we found that there was decreased accuracy to congruently gripped non-objects. The results indicate that motor-based affordance, based on whether stimuli are depicted with a congruent grip, can spill-over to affect categorization responses either positively (when the familiar affordance aligns with the required behavioral response) or negatively (when the affordance mis-matches the behavioral response).

### Conflict of interest statement

The authors declare that the research was conducted in the absence of any commercial or financial relationships that could be construed as a potential conflict of interest.
